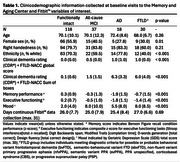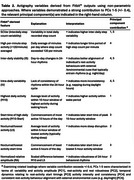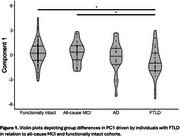# 24‐hour rest‐activity rhythms differ across Alzheimer's disease and related dementias

**DOI:** 10.1002/alz70857_097780

**Published:** 2025-12-24

**Authors:** Emilie V. Brotherhood, Coty Chen, Claire J. Cadwallader, Isabel Sible, Anna M. VandeBunte, Rowan Saloner, Linh Pham, Valentina E. Diaz, Adam M. Staffaroni, Joel H Kramer, Kaitlin B Casaletto, Emily W. Paolillo

**Affiliations:** ^1^ Memory and Aging Center, UCSF Weill Institute for Neurosciences, University of California, San Francisco, San Francisco, CA, USA; ^2^ Global Brain Health Institute, University of California San Francisco, San Francisco, CA, USA

## Abstract

**Background:**

Passive actigraphy monitoring via commercial wearable devices offers a scalable opportunity to capture real‐world changes in sleep‐wake cycles affected in Alzheimer's disease and related dementias (ADRD). We characterized continuous rest‐activity patterns derived from Fitbit™ data, and examined differences between healthy adults and ADRD cohorts.

**Method:**

Tri‐axial actigraphy data (step counts per minute), clinicodemographic information, cognitive and functional performance, and mood scores were completed at baseline in functionally intact adults (*N* = 118), individuals with single/multi‐domain mild cognitive impairment (all‐cause MCI; *N* = 37), Alzheimer's disease dementia (AD; *N* = 18); or frontotemporal lobar degeneration (FTLD, *N* = 30; see Table 1). Activity patterns were quantified by calculating rest‐activity aggregates (e.g. averaged step count variability) and features derived from minute‐level step count data (see Table 2). Principal component analysis (PCA) was performed for data reduction of nine features using singular value decomposition (SVD). Five components (accounting for >85% of the total variance in rest‐activity patterns) were characterized according to patterns of strong variable contributions (+/‐ 0.4; see Table 2). Principal components (PCs) 1‐5 were subsequently examined for associations with measures of cognitive and functional decline. The first of these components was further analyzed for group differences with the objective of characterizing syndrome‐specific 24‐hour activity rhythms.

**Result:**

Controlling for participant age and biological sex, PC1 [activity variability and amplitude] was negatively associated with both CDR®+FTLD‐NACC sum of boxes score [β=‐0.30, s.e.=0.15 *p* = 0.010] and CDR®+FTLD‐NACC global score [β=‐0.06, s.e.=0.03, *p* = 0.010]). Dynamics relating to rest‐activity start timing [PC3] was also negatively associated with CDR®+FTLD‐NACC global score [β=‐0.06, s.e.=0.04, *p* = 0.040]). PC1 also revealed diagnostic group differences (ηp^2^ = 0.06, *p* = 0.004). Post hoc Tukey HSD analysis indicated that this omnibus group difference was driven by a reduction in FTLD‐associated group PC1 scores relative to both functionally intact (mean difference=‐1.05, *p* = 0.004) and all‐cause MCI cohorts (mean difference= ‐1.09, *p* = 0.019; see Figure 1).

**Conclusion:**

Activity variability and amplitude derived from Fitbit™ data revealed distinct activity profile behaviors in individuals diagnosed with FTLD‐associated syndromes, which differed from individuals living with mild cognitive impairment or individuals who are functionally intact.